# Supplementary material for: A thiol probe for measuring unfolded protein load and proteostasis in cells
Source: Nat Commun. 2017 Sep 7;8:474. doi: 10.1038/s41467-017-00203-5 (PMC5589734; doi:10.1038/s41467-017-00203-5)
Supplement: Supplementary file 1 — Supplementary Information [file 41467_2017_203_MOESM1_ESM.pdf]

File Name: Supplementary Information
Description: Supplementary Figures and Supplementary Table

File Name: Peer Review File
Description:

File Name: Supplementary Dataset 1
Description: Detailed statistical summary of the data in the Figures.

File Name: Supplementary Dataset 2
Description: Description of the proteins/peptides discovered by proteomics of TPE-MI treated tunicamycin stress and control samples. Relates to Figure 6.

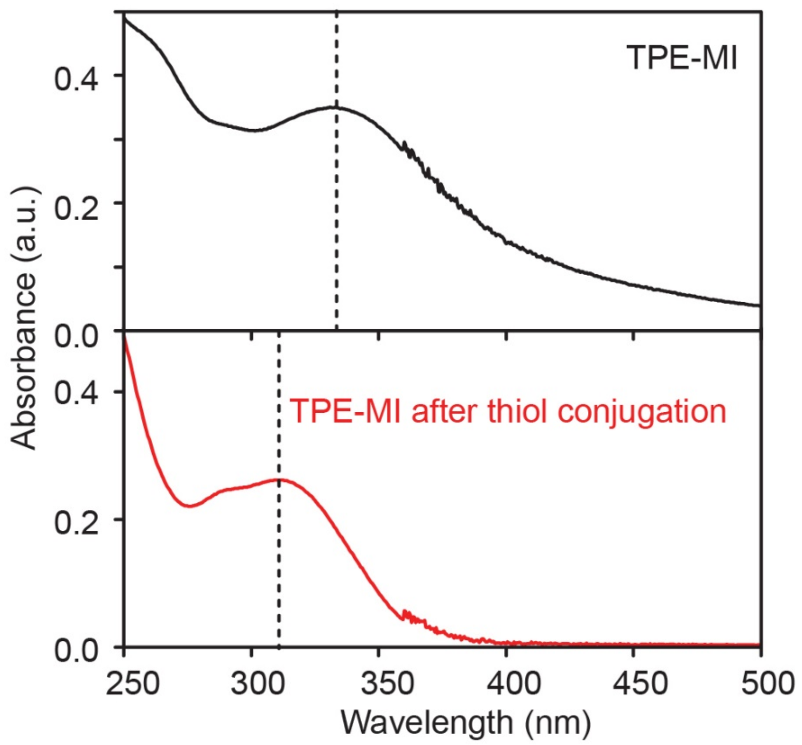

Supplementary Figure 1: Change of TPE-MI conjugation by reaction with thiol. Absorbance spectrum of TPE-MI before and after reaction with glutathione in PBS.

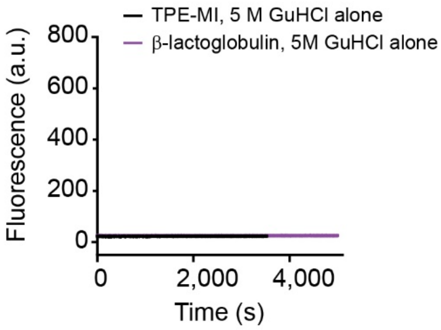

Supplementary Figure 2: Control data corresponding to Fig. 2a. Timecourse of fluorescence of TPE-MI in denaturant, and β-galactosidase alone in denaturant.

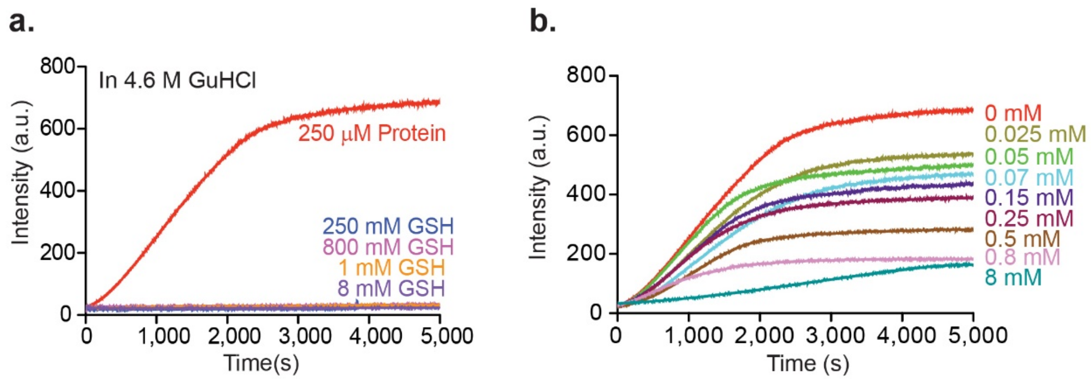

Supplementary Figure 3: Response of TPE-MI to GSH. Time-course fluorescence intensity of 50 μM TPE-MI (**a**) with different concentrations of GSH alone versus β-lactoglobulin (250 μM) alone as a reference and (**b**) in the presence of β-lactoglobulin (250 μM) and GSH (0–8 mM) in 4.6 M GuHCl solution.

a.

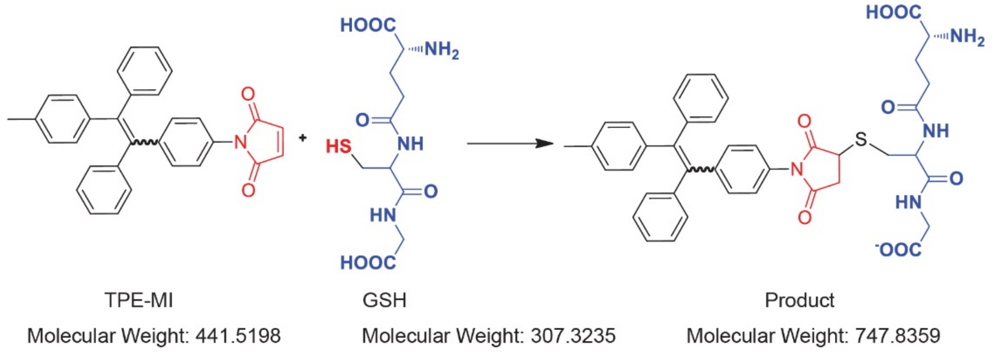

b.

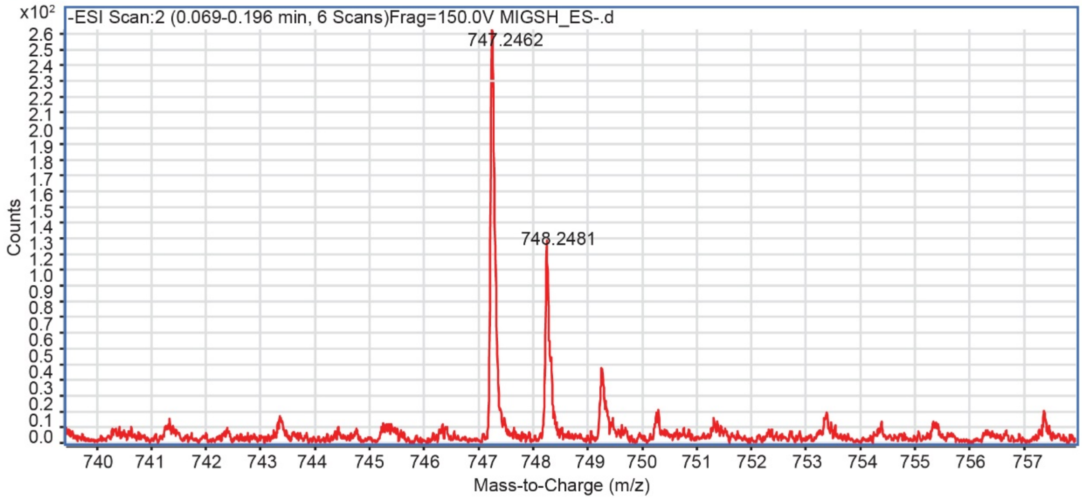

Supplementary Figure 4: TPE-MI conjugates to GSH. (**a**) Reaction scheme and (**b**) mass spectrum of the GSH-TPE-MI conjugate.

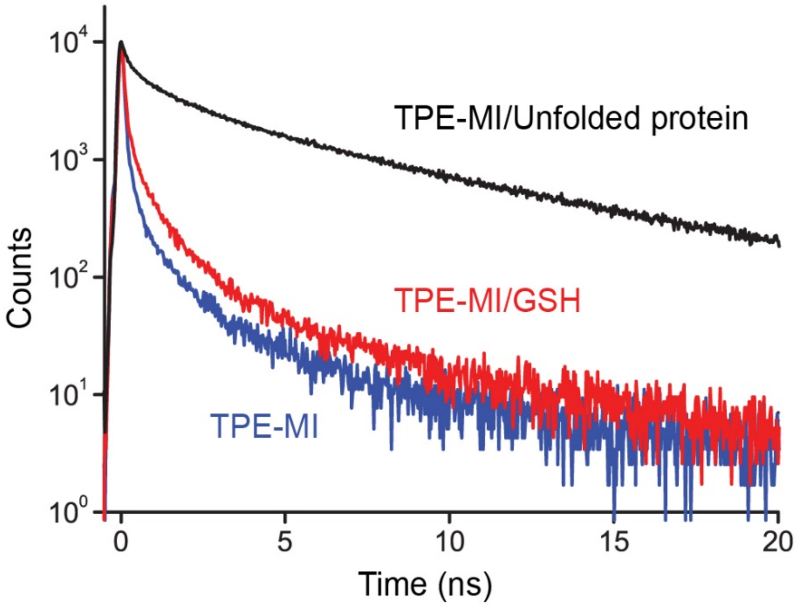

Supplementary Figure 5: Fluorescence lifetime indicates the rigidity of the environment. Fluorescence decay traces of TPE-MI (50 μM) with/without β-lactoglobulin (250 μM) in 4.6 M GuHCl or GSH (250 μM).

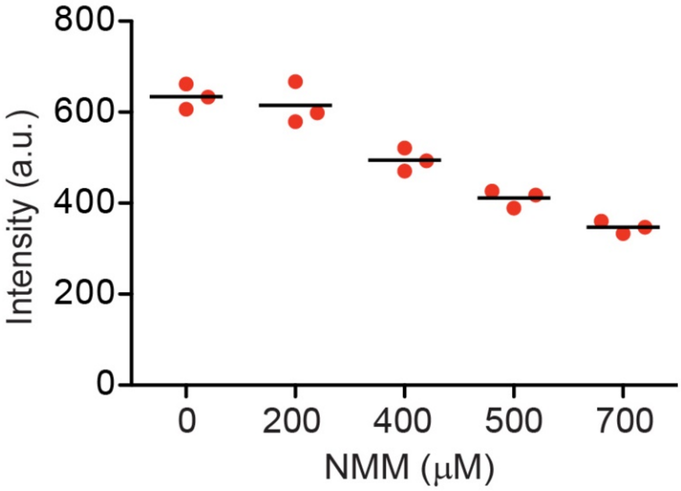

Supplementary Figure 6: Testing the specificity of TPE-MI to intracellular thiols using N-methylmaleimide (NMM) pretreatment. Neuro-2a cells were pretreated with shown concentrations of thiol-modifying agent NMM prior to TPE-MI treatemnt**.** Means and replicates shown.

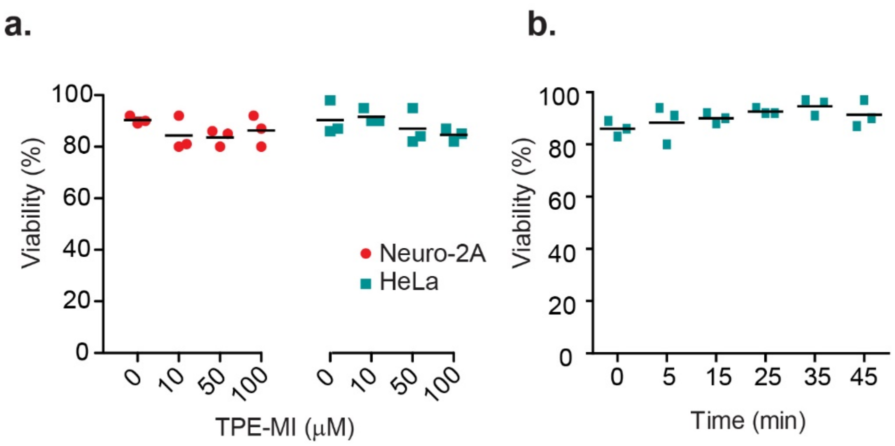

Supplementary Figure 7: Cytotoxicity test of TPE-MI in HeLa and Neuro-2a cell-lines. (**a**) Cell viability was measured with Trypan Blue reactivity in an automated cell counter. 3 replicates plotted with mean are shown for cells 30 min after treatment with TPE-MI. (**b**) Time-dependence on viability after treatment of Neuro-2a cells with 100 μM TPE-MI. Means and replicates shown.

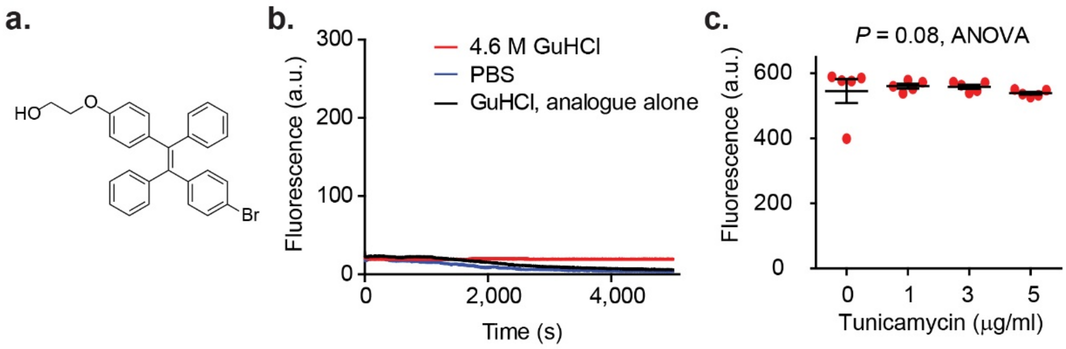

Supplementary Figure 8: Lack of reactivity of a non-alkylating analogue of TPE-MI with unfolded proteins. (**a**) Structure of a TPE analogue with no cysteine reactive site — the MI group was replaced with bromine and the hydroxyethyl group was added to improve the solubility and cell permeability. (**b**) Assay of the analogue with β-lactoglobulin control protein shows no reactivity. (**c**) The analogue shows no change in fluorescence over background on Neuro2a cells when treated with ER stressor tunicamycin using the same conditions as in Fig 5. Bars show mean ± S.E.M. Full details of the statistics are shown in Supplementary Data 1.

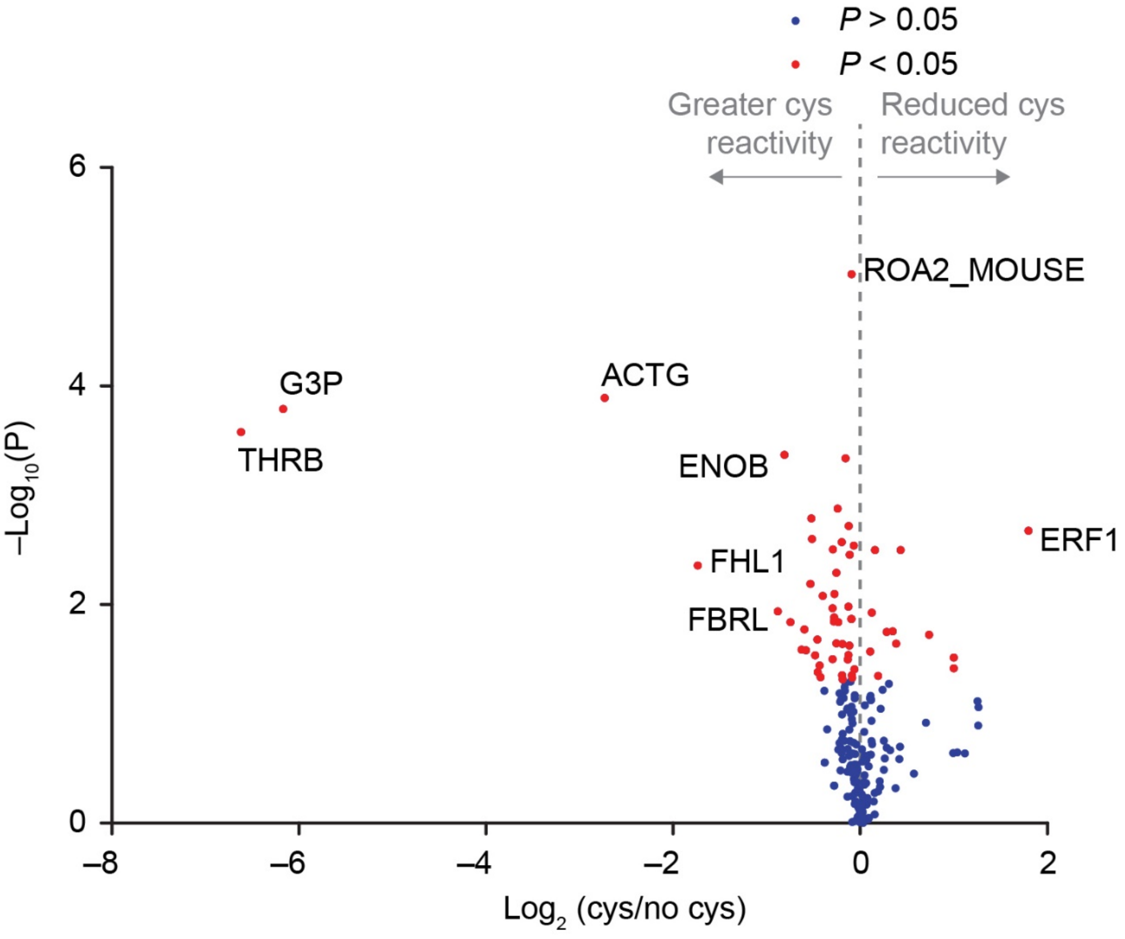

Supplementary Figure 9: Volcano plot of changes in cysteine reactivity attributable to tunicamycin treatment. Relates to Fig. 6b. Shown are the peptide abundance ratios for cys-containing peptides between tunicamycin and control conditions. *P* values were calculated using the Student's t-test for 5 biological replicates.

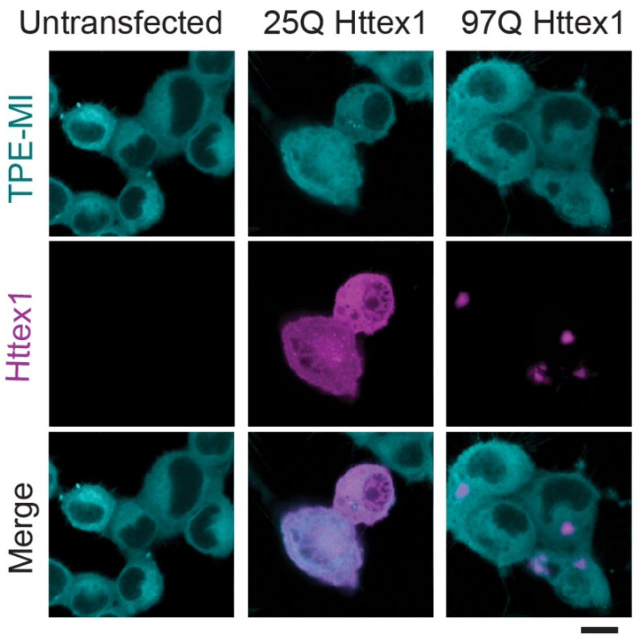

Supplementary Figure 10: Confocal images of Neuro-2a cells with Httex1 expression after TPE-MI staining. Images shows fluorescence of mCherry-tagged 25QHttex1 and 97QHttex1 and TPE-MI fluorescence. Scale bar, 20 µm.

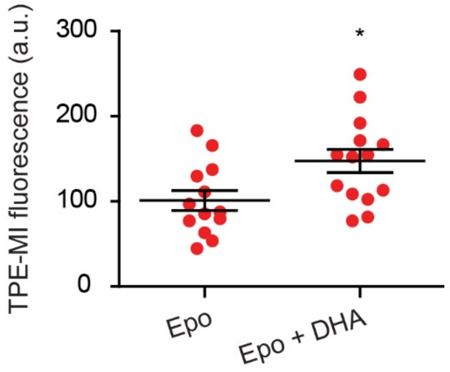

Supplementary Figure 11. TPE reports on accumulation of unfolded proteins in malaria parasites (*P. falciparum*) treated with dihydroartemisinin (DHA) and epoxomicin. Relates to Fig. 8. TPE-MI fluorescence values from individual trophozoites within red blood cells treated with or without DHA in the presence of epoxomicin measured by confocal microscopy. Bars represent the mean ± S.E.M. * represents a significant difference. The details of the statistics in this figure are provided in Supplementary Data 1.

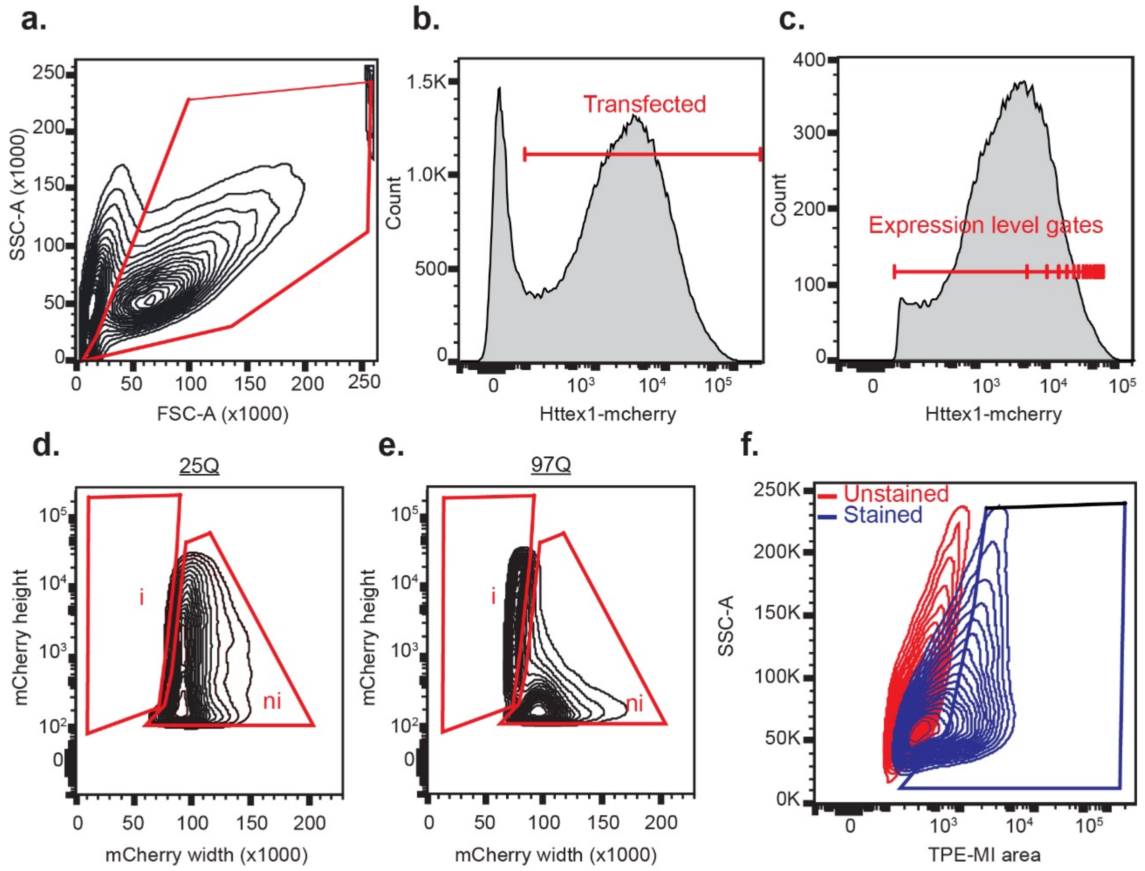

Supplementary Figure 12: Flow cytometry gating strategy. Representative gates are shown for (**a**) side and forward side scattering gates to focus on the viable cell population, (**b**) transfected cell population focusing on cells with mCherry fluorescence, (**c**) the bins for different expression levels based on Httex1-mCherry fluorescence, (**d**) and (**e**) pulse shape analysis (PulSA) of the Httex1-mCherry fluorescence used to mark cells with inclusions (i) from cells without inclusions (ni), (**f**) Cells containing higher than background TPE-MI fluorescence.

Supplementary Table 1: Fluorescence decay parameters of TPE-MI.

Entry	species[a]	A_1[b]	A_2[b]	τ_1 [ns][b]	τ_2 [ns][b]	$\langle\tau\rangle$ [ns][c]
1	TPE-MI/Unfolded protein	30.7	69.3	1.283	6.121	4.636
2	TPE-MI/GSH	51.1	48.9	0.599	3.524	2.029
3	TPE-MI	100	0	0.067	0	0.067

[a] [TPE-MI] = 5 μM; [protein] = [GSH] = 25 μM; [GuHCl] = 4.6 M. [b] Determined from eq. 1, where *A* and *τ* are the fractional amount and fluorescence lifetime of different species, respectively. [c] Weighted mean lifetime determined from eq. 2.

$$I = A_1 e^{-t/\tau_1} + A_2 e^{-t/\tau_2} \quad (1)$$

$$\langle\tau\rangle = \frac{A_1\tau_1 + A_2\tau_2}{A_1 + A_2} \quad (2)$$
